# Traffic Management in IoT Backbone Networks Using GNN and MAB with SDN Orchestration

**DOI:** 10.3390/s23167091

**Published:** 2023-08-10

**Authors:** Yanmin Guo, Yu Wang, Faheem Khan, Abdullah A. Al-Atawi, Abdulwahid Al Abdulwahid, Youngmoon Lee, Bhaskar Marapelli

**Affiliations:** 1 Shandong Research Institute of Industrial Technology, Jinan 250061, China; guoyanmin@sriit.cn (Y.G.); wangyu@sriit.cn (Y.W.); 2 Department of Computer Engineering, Gachon University, Seongnam-si 13120, Republic of Korea; 3 Department of Computer Science, Applied College, University of Tabuk, Tabuk 47512, Saudi Arabia; a.alatawi@ut.edu.sa; 4 Department of Computer and Information Technology, Jubail Industrial College, Royal Commission for Jubail and Yanbu, Jubail Industrial City 31961, Saudi Arabia; abdulwahida@rcjy.edu.sa; 5 Department of Robotics, Hanyang University, Ansan 15588, Republic of Korea; 6 Department of Computer Science and Information Technology, KL Deemed to be University (KLEF), Vijayawada 522502, AP, India; bhaskarmarapelli@gmail.com

**Keywords:** traffic management, anomaly detection, intrusion detection, network security, internet of things, network traffic analysis, machine learning, SDN (software-defined networking), GNN (graph neural network), MAB (multi-armed bandit)

## Abstract

Traffic management is a critical task in software-defined IoT networks (SDN-IoTs) to efficiently manage network resources and ensure Quality of Service (QoS) for end-users. However, traditional traffic management approaches based on queuing theory or static policies may not be effective due to the dynamic and unpredictable nature of network traffic. In this paper, we propose a novel approach that leverages Graph Neural Networks (GNNs) and multi-arm bandit algorithms to dynamically optimize traffic management policies based on real-time network traffic patterns. Specifically, our approach uses a GNN model to learn and predict network traffic patterns and a multi-arm bandit algorithm to optimize traffic management policies based on these predictions. We evaluate the proposed approach on three different datasets, including a simulated corporate network (KDD Cup 1999), a collection of network traffic traces (CAIDA), and a simulated network environment with both normal and malicious traffic (NSL-KDD). The results demonstrate that our approach outperforms other state-of-the-art traffic management methods, achieving higher throughput, lower packet loss, and lower delay, while effectively detecting anomalous traffic patterns. The proposed approach offers a promising solution to traffic management in SDNs, enabling efficient resource management and QoS assurance.

## 1. Introduction

The increasing demand for high-bandwidth applications, such as video streaming, online gaming, and cloud computing, has resulted in a surge of network traffic. However, the growth in network traffic is often accompanied by network congestion, which can significantly impact network performance and user experience. One approach to mitigating network congestion is traffic management, a technique that prioritizes, limits, or blocks specific types of traffic based on predefined policies [1].

While traffic management has proven to be effective in managing network congestion, it faces several challenges in modern networks. First, the growing complexity of network infrastructure and traffic patterns requires more intelligent and adaptive traffic-shaping techniques to achieve optimal performance [2]. Second, traditional traffic-shaping approaches often rely on static policies, which may not be able to adapt to changing network conditions or user demands. Finally, the limited visibility into network traffic and topology makes it difficult to accurately identify and prioritize critical traffic flows. Typically, IoT gateways are employed to connect IoT servers through either cellular or non-cellular connections. These gateways gather IoT traffic within their respective coverage areas and transfer the accumulated data to the server using an optical network called IoT backhaul. The quantity of traffic generated by a large number of IoT devices is significant, despite each individual device producing only a small amount of traffic. One way to manage this traffic is by using an IoT backhaul network that consists of Layer-2 or Layer-3 aggregation switches. Such a network can handle IoT traffic effectively by utilizing statistical multiplexing while remaining cost-effective [3].

In addition, the growth of the Industrial Internet has brought about significant challenges in managing and regulating the massive amounts of data generated by the multitude of sensors in factories [4]. These sensors collect real-time and non-real-time data, and the transmission of this data is subject to high demands and periodicity, resulting in sudden changes in traffic rates. These changes can lead to the waste of bandwidth resources and network congestion. While aggregated switch networks have been employed to control IoT traffic [5], they may encounter partial traffic loss and bursts that necessitate traffic management methods. Nevertheless, conventional traffic management techniques have constraints on their effectiveness, and there has been a recommendation for employing cooperative traffic management using multiple switches as a solution [6]. In addition, IoT gateways can lead to congestion by producing a massive access problem (MAP) [7], which can be alleviated by employing the quasi-deterministic transmission policy (QDTP) traffic management method [7]. This method prioritizes real-time traffic over non-real-time traffic and enhances connectivity capacity while regulating non-real-time packet flows. Several input rate control techniques have been proposed in the literature to enhance the smoothing and regulation effects of input traffic, particularly for hard real-time traffic (HRT) and soft real-time traffic (SRT). The challenges posed by managing traffic in the Industrial Internet demand effective and efficient traffic management techniques that can handle the sudden changes in traffic rates while optimizing network performance and reducing congestion.

To address these challenges, there has been growing interest in applying machine learning techniques to traffic management [8]. In particular, the individual Graph Neural Networks (GNNs) [9,10], Multi-Armed Bandit (MAB) algorithms, and Software-Defined Networking (SDN) [11] has shown great potential in providing a flexible and powerful solution for managing traffic in complex networks. GNNs can learn from the complex network structure and traffic patterns, while MAB algorithms can adaptively explore different shaping configurations. SDN can provide fine-grained control over traffic flow within the network, enabling dynamic policy updates based on real-time traffic conditions.

**Figure 1:**
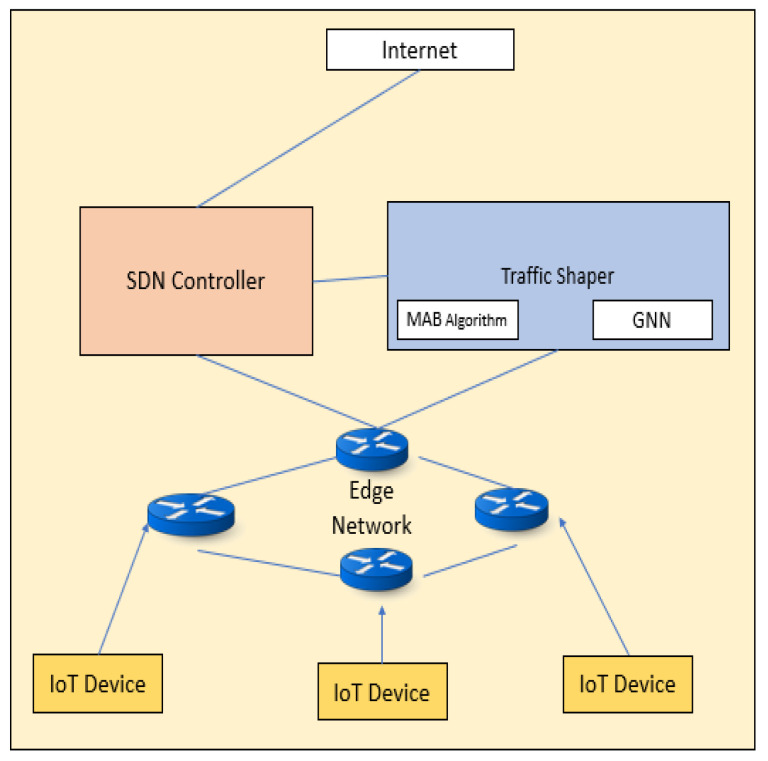
Proposed system Architecture.

This paper proposes an approach that combines GNN, MAB, and SDN for traffic management in IoT networks, Figure 1 shows the Proposed system Architecture. We demonstrate the effectiveness of our proposed approach using real-world traffic data and evaluate its performance in comparison to traditional traffic management techniques. We also discuss the design and implementation considerations for deploying such a system in real-world networks. Our results show that the proposed approach can significantly improve network performance and reduce congestion, highlighting the potential of machine learning in shaping the future of traffic management.

## 2. Literature Review

### 2.1. SDN Based Traffic Management

SDN was initially developed by Nicira Networks, utilizing earlier research from various universities. Its objective is to facilitate open and user-driven control of network hardware, thereby increasing the proximity of applications and devices while segregating control and data planes. Example from Ali, Jehad et al. [12] SDN is used to address the mobility problem, Rani, Shalli et al. [13] proapsed SDN and blockchain technology framework to enhance security, reduce response time, and improve scalability by detecting potential attacks with SDN and transmitting secured data to the blockchain.

Traditional networks have a rigid linkage between control and data planes, which SDN overcomes by separating the two, resulting in better controllability, security [14], and network resource optimization. OpenFlow is presently the most prevalent SDN protocol, with its design specifications.

In [15], the authors address the challenge of handoff delay and failure in SDN-based IEEE 802.11 networks. They propose a hybrid clustering technique and distributed mobility management, demonstrating the potential of SDN in improving network performance and mobility management. Next, Lei and Kai et al. [16] focus on congestion control in SDN using deep reinforcement learning and multi-task learning. This study highlights the potential benefits of integrating ML techniques in SDN to optimize network performance and traffic management. Lonare and Mahesh B. et al. [17] present a dynamic framework, D-SAVI, for lightweight Source Address Validation (SAVI) in SDN. It showcases the potential of SDN in enhancing network security and reducing resource consumption through lightweight SAVI implementations. Al Mtawa and Yaser et al. [18] explore the integration of AI and big data technologies with SDN for intelligent network control and traffic optimization. This study emphasizes the potential benefits of leveraging SDN and AI in achieving intelligent network control and enhancing traffic efficiency. Guo and Aipeng et al. [19] investigate the impact of failover on SDN and conventional networks. It highlights the resilience of SDN-based network management and its superiority in handling disruptions compared to conventional networks. Subardono and Alif et al. [20] explore SDN-based network management integrated with load balancing and failover mechanisms. It emphasizes the effectiveness of SDN in network efficiency optimization and network availability through load balancing and failover techniques. Abar and Tasnim [21] address the challenges of SAVI deployment in SDN and propose a dynamic framework, D-SAVI, for lightweight SAVI. This work highlights the potential of SDN in addressing complexity and performance costs associated with SAVI while maintaining network security. Finally, in [22], the authors propose work on DTM (Dynamic mechanism for Traffic Management), a promising energy-aware dynamic mechanism for traffic management. By leveraging the SDN paradigm, DTM effectively adjusts network resources to prevent over-provisioning and reduce power consumption during low-demand periods without compromising the quality of service.

In the above, we reviewed literature that collectively demonstrates the significant role of SDN in network management, encompassing various aspects such as mobility management, congestion control, security, network availability, and efficiency optimization.

### 2.2. SDN and Machine Learning-Based Traffic Management

In the realm of SDN and machine learning for traffic management, researchers have explored various approaches to address different challenges. Authors in [23,24] highlight the significance of ML in enhancing security in SDN-based networks, with a focus on detecting DDoS attacks and virulent traffic during congestion. Moving beyond security, Ben Letaifa and Asma [25] discuss the application of SDN + ML in optimizing video streaming for improved user experience. Meanwhile, Filali and Abderrahime et al. [26] tackle the load balancing problem in SDN networks to support low-latency communications.

In the realm of congestion control, Lei and Kai et al. [16] introduce a multi-task ML model for congestion control and load balancing, showcasing the potential of SDN + ML in efficiently managing network traffic. Furthermore, Akbar and Aamir et al. [27] explore ML-based traffic optimization in SDN, specifically focusing on predicting user QoE. These papers collectively demonstrate the versatility of SDN + ML in addressing various network demands.

Security remains a key concern in SDN environments, as highlighted in a paper by Nadeem and Muhammad Waqas et al. [28]. The paper evaluates feature selection methods for ML-based DDoS detection, emphasizing the importance of mitigating attacks to ensure network resilience. Similarly, in paper [29] emphasizes the significance of accurate topology classification in SDN architectures and explores the performance of supervised ML algorithms in classifying network topologies.

In the paper by Ramya and Manoharan et al. [30], the authors present an exciting and innovative approach that combines Software-Defined Networking (SDN) and Machine Learning (ML) to address network traffic management challenges. The study aims to predict the optimal number of controllers required for network operations by integrating SDN with Network Function Virtualization (NFV) and ML techniques. To achieve this, they deploy a centralized ML-based prediction mechanism as a Virtual Network Function (VNF) in the NFV environment, paving the way for more efficient and automated network management. The utilization of the K-Medoid algorithm for optimal controller placement further strengthens the proposal’s practicality, enhancing the effectiveness and scalability of the approach. This integration of SDN, NFV, and ML techniques demonstrates the potential for intelligent and adaptable network management solutions, which can significantly improve network performance and resource utilization in dynamic and ever-changing environments.

Lastly, Zitouna and Imene Elloumi [31] present an intelligent orchestrator for Open vSwitch in SDN, showcasing the integration of ML techniques. The paper addresses tasks such as information extraction, reinforcement learning for destination identification, and supervised learning for intelligent SDN controller selection. Collectively, these studies demonstrate the potential of SDN + ML in enabling intelligent network management and optimization.

The proposed work of GNN + MAB + SDN holds significant importance compared to both traditional SDN and the emerging field of SDN + ML. While SDN offers a paradigm shift in network management and control, and SDN + ML leverages machine learning techniques for enhanced performance, the proposed GNN + MAB + SDN approach takes it a step further by integrating graph neural networks (GNNs) and multi-armed bandit (MAB) algorithms into the SDN framework.

The incorporation of GNNs in the proposed work allows for the effective capture of complex relationships and dependencies within the network, leading to more accurate and efficient decision-making. GNNs have demonstrated high effectiveness in modeling graph structures and extracting meaningful features, making them well-suited for analyzing and optimizing network traffic patterns.

Additionally, the integration of MAB algorithms introduces a dynamic and adaptive element to the SDN framework. By learning from past experiences and exploring different options, MAB algorithms empower the system to make informed decisions on resource allocation, traffic routing, and load balancing in real-time. This adaptive approach significantly enhances the overall performance, scalability, and resource utilization of the network.

Compared to traditional SDN, which relies on pre-defined rules and static decision-making, the proposed GNN + MAB + SDN approach offers a more intelligent, flexible, and responsive network management system. It leverages the power of machine learning and optimization algorithms to optimize network traffic, reduce congestion, improve Quality of Service (QoS), and adapt to dynamic network conditions. This holistic approach aligns with the growing demands of modern networks, where scalability, efficiency, and adaptability are crucial for meeting the diverse needs of applications and users.

In conclusion, the proposed approach of GNN + MAB + SDN presents a promising advancement in the field of network management by combining the capabilities of graph neural networks, multi-armed bandit algorithms, and software-defined networking. It offers a more intelligent, adaptive, and efficient approach to traffic management compared to traditional SDN and even the emerging field of SDN + ML. This work has the potential to significantly enhance the performance, reliability, and scalability of network infrastructures in various domains, paving the way for future advancements in network management and optimization. For a comprehensive overview, Table 1 presents a comparison of the existing and proposed models.

## 3. Proposed Model

### 3.1. Traffic Management

Traffic management is a critical step in optimizing network performance by controlling the rate of data transmission. It can help to manage congestion and reduce packet loss, improving the overall quality of service (QoS) for end-users. In this section, we will discuss the traffic management component of the proposed model.

Traffic management involves regulating the flow of data through the network by introducing delays and buffering packets. This is typically achieved using a token bucket algorithm, where tokens are generated at a fixed rate and consumed by packets as they are transmitted. If the bucket is empty, packets are queued until sufficient tokens are available.

The token bucket algorithm can be represented mathematically as follows:

At time *t*, the number of tokens in the bucket can be calculated as:

(1) $$B\left(t\right)=\min\left(B\left(t-1\right)+r\left(t-t_{last}\right),B_{max}\right)$$

where B(t−1) is the number of tokens at the previous time step, *r* is the token generation rate, *t* is the current time, $t_{last}$ is the time when the last token was generated, and $B_{max}$ is the maximum bucket size.

When a packet of size *P* arrives at time *t*, it is immediately transmitted if there are enough tokens available in the bucket:

(2) ifB(t)≥P,thentransmitthepacket

Otherwise, the packet is queued until sufficient tokens become available:

ifB(t)<P,thenaddthepackettothequeue. Packets in the queue are transmitted in order of arrival as soon as sufficient tokens become available.

The token bucket algorithm [32] can be further optimized by adjusting the token generation rate based on network conditions. For example, if congestion is detected, the token generation rate can be reduced to prevent further congestion.

**Figure 2:**
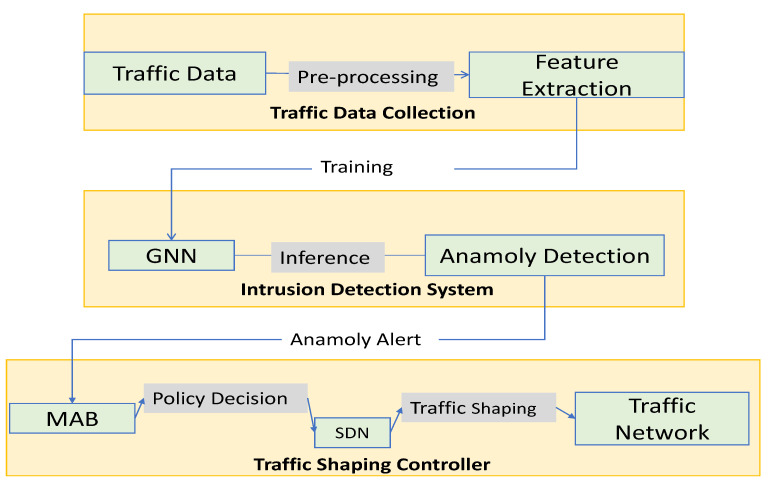
Data flow in the proposed system.

The Data flow Figure 2 of the proposed starts with the input data, which is passed through a feature extraction module to extract relevant features that will be used for traffic management. The extracted features are then passed to the GNN module, which is responsible for learning the complex patterns in the traffic data and predicting the optimal traffic-shaping policy.

The predicted policy is then sent to the SDN controller, which is responsible for configuring the network to apply the policy. The SDN controller communicates with the network switches through the OpenFlow protocol, which allows it to modify the forwarding rules on the switches based on the predicted policy.

The network switches forward the traffic according to the configured policy, and a traffic monitoring module monitors the traffic. The monitoring module collects traffic statistics, which are then used by the MAB module to update the policy selection strategy. The MAB module uses a multi-armed bandit algorithm to balance exploration and exploitation and to select the optimal policy based on the current traffic conditions.

Overall, the proposed architecture leverages the strengths of SDN, GNN, and MAB to create an adaptive traffic management system that can optimize network performance in real-time based on the current traffic conditions.

Overall, traffic management is an important component of the proposed model for optimizing network performance and ensuring a high quality of service for end-users.

### 3.2. GNN for Understanding Traffic Pattern

Graph Neural Networks (GNNs) are a type of deep learning method that have recently gained popularity in the field of traffic analysis and prediction. GNNs are particularly effective in modeling and analyzing data that can be represented as graphs, which makes them well-suited for analyzing traffic patterns.

The basic idea behind GNNs is to learn a set of node and edge embeddings that capture the underlying structure of the graph. These embeddings can then be used to perform various downstream tasks, such as node classification, edge prediction, or graph clustering.

In the context of traffic analysis, GNNs can be used to model traffic flow data as a graph, where each node represents a road segment or intersection and each edge represents the flow of traffic between them. The GNN can then learn a set of embeddings that capture the underlying patterns of traffic flow, such as congestion, bottlenecking, and routing preferences.

The mathematical equations used in GNNs are typically based on message-passing algorithms, which allow nodes in the graph to communicate and update their embeddings based on the embeddings of their neighbours. One commonly used message-passing algorithm is the Graph Convolutional Network (GCN), which is based on the following equation:

(3) $$\left\{\begin{array}{c} h_{i}^{\left(l+1\right)}=\sigma \left(\sum_{j\in N(i)}\frac{1}{c_{ij}}W^{\left(l\right)}h_{j}^{\left(l\right)}\right) \end{array}\right.$$

where $h_{i}^{\left(l\right)}$ represents the embedding of node *i* in layer *l*, σ(·) is an activation function, N(i) is the set of neighbours of node *i*, $W^{\left(l\right)}$ is a learnable weight matrix for layer *l*, and $c_{ij}$ is a normalization constant that depends on the degree of nodes *i* and *j*.

This equation captures the idea of passing messages from neighbouring nodes to update each node’s embedding, while also incorporating a normalization term to account for differences in node degree. By stacking multiple layers of GCNs, the GNN can learn increasingly complex representations of the graph, which can be used for downstream tasks.

### 3.3. GNN Architecture in the Proposed Approach

**Figure 3:**
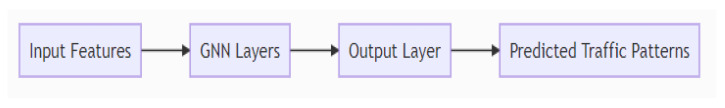
GNN architecture in the proposed work.

The proposed Graph Neural Network (GNN) architecture takes into account the node features, edge connections, and graph structure through multiple layers of graph convolutional units. Each graph convolutional unit propagates information through the graph, leveraging the specified neighbourhood relations and performing calculations on node and edge features. In our specific approach (see Figure 3), we employ a three-layer graph convolutional network (GCN) with 64 hidden units as part of the GNN architecture. The input to the GNN is a graph representation of the network traffic data, where each node represents a traffic flow, and each edge signifies the connection between flows. By updating the node representations iteratively using data from neighbouring nodes, the GNN learns patterns and relationships in the traffic data. To enhance the model’s expressiveness, we include activation functions such as ReLU in the GNN architecture, introducing non-linearity. Moreover, we incorporate dropout regularization to reduce overfitting and improve generalization efficiency. It is essential to note that Algorithm 1 describes the GNN’s learning procedure, whereas the overall architecture of the model comprises its layers, units, activation functions, and regularization strategies. While Algorithm 1 provides a general overview of how the GNN updates its parameters during training to minimize loss and enhance prediction accuracy for traffic patterns, the complete GNN architecture is designed to efficiently analyze and represent complex network traffic behavior.
**Algorithm 1** GNN for Understanding Traffic Patterns.**Require:** Traffic dataset *D* with traffic flow information, adjacency matrix *A*, number of graph convolution layers *L*, and number of output classes *K*.**Ensure:** Trained GNN model $f_{\theta }\left(X\right)$ for predicting traffic patterns. 1:Construct input graph G=(V,E) from *D* and *A* 2:Initialize node feature matrix $X^{\left(0\right)}\in R^{n\times d}$, where *n* is the number of nodes and *d* is the dimension of the node features 3:**for** l=1 to *L* **do** 4:    Compute node embeddings using graph convolution layer: $H^{\left(l\right)}=\sigma \left(\tilde{A}X^{\left(l-1\right)}W^{\left(l\right)}\right)$ 5:    Update node feature matrix: $X^{\left(l\right)}=H^{\left(l\right)}$ 6:**end for** 7:Compute final node embeddings: $Z=\mathrm{mean}(H^{\left(L\right)},axis=1)$ 8:Predict output classes: $\hat{Y}=\mathrm{softmax}\left(ZW^{\left(f\right)}\right)$ 9:Compute loss function: $J\left(\theta \right)=-\frac{1}{m}\sum_{i=1}^{m}\sum_{k=1}^{K}Y_{i,k}\log\hat{Y}i,k$ 10:Update model parameters using backpropagation: θ←θ−α∇θJ(θ) 11:Repeat steps 2–9 until convergence or maximum number of epochs is reached.

In summary, GNNs are a powerful tool for analyzing traffic patterns by modelling traffic flow data as a graph and learning embeddings that capture the underlying patterns of traffic flow. The mathematical equations used in GNNs are based on message-passing algorithms, such as the GCN, which allow nodes to communicate and update their embeddings based on the embeddings of their neighbours.

The Table 2 provides a clear and concise explanation of each term used in the algorithm, helping to clarify their meanings and roles within the context of the GNN model for understanding traffic patterns.

In this algorithm, we first construct an input graph from the traffic dataset and adjacency matrix. We then initialize the node feature matrix and apply a specified number of graph convolution layers to compute node embeddings. The final node embeddings are aggregated to form a graph-level representation, which is used to predict output classes using a softmax layer. We then compute the cross-entropy loss function and use backpropagation to update the model parameters. Finally, we repeat the process until convergence or a maximum number of epochs is reached.

The dataset, D, used for modeling the Graph Neural Network (GNN) in the proposed approach contains a wide range of crucial features, contributing to a comprehensive understanding of network behavior and optimizing traffic management decisions. These features encompass various aspects, each serving a specific purpose in network analysis. The duration feature provides insights into the temporal aspects of network flows, allowing the identification of short-lived or long-lasting connections. The protocol type and service features offer information about the nature of network communication, distinguishing between different protocols and services, such as HTTP, FTP, or VoIP. Source and destination IP addresses, along with ports, play a fundamental role in routing decisions, ensuring traffic is directed to the appropriate endpoints. Packet size and traffic volume indicate the amount of data transmitted, influencing bandwidth allocation and capacity planning. Considering different service requirements and quality of service guarantees, traffic rate, priority, and traffic class features enable the appropriate treatment of various types of traffic. Additionally, analyzing traffic patterns helps detect recurring patterns or sudden bursts, enabling effective congestion management and resource allocation. Quality of service parameters, such as delay, jitter, and packet loss, provide insights into network performance, facilitating the optimization of traffic management policies to meet desired performance targets. Finally, the network topology feature offers a comprehensive understanding of the network structure, encompassing switches, routers, and links. This information is crucial for efficient traffic routing and resource utilization. Combining these diverse features provides a holistic view of network traffic characteristics, enabling the GNN model to capture and analyze the complex dynamics of the network environment effectively. With this rich dataset, the proposed approach gains valuable insights into network behavior, leading to more informed and optimized traffic management decisions for enhanced network performance.

**Table 2:** Explanation of terms.

Term	Explanation
*D*	Traffic dataset containing information about traffic flows.
*A*	Adjacency matrix representing the relationships between traffic flows.
*L*	Number of graph convolution layers.
*K*	Number of output classes.
G=(V,E)	Input graph constructed from the traffic dataset and adjacency matrix.
$X^{\left(0\right)}$	Node feature matrix representing the initial features of the nodes.
$H^{\left(l\right)}$	Node embeddings computed using the graph convolution layer at the *l*-th layer.
$\tilde{A}$	Normalized adjacency matrix.
$W^{\left(l\right)}$	Weight matrix for the graph convolution layer at the *l*-th layer.
*Z*	Final node embeddings obtained by taking the mean of the embeddings from the last layer.
$\hat{Y}$	Predicted output classes generated by applying softmax to the final node embeddings.
J(θ)	Loss function that quantifies the difference between predicted output classes and ground truth labels.
α	Learning rate determining the step size for updating model parameters.
θ	Model parameters to be updated during training.

Note that in Algorithm 2, we use the GNN output θ to calculate the expected reward for each arm at each time step, and update the probability distribution of the arms using the UCB algorithm. The chosen action at each time step is then the one with the highest probability in the updated distribution. The multi-arm bandit algorithm for traffic management that utilizes the output of GNN for understanding traffic patterns involves several steps. First, the GNN is used to analyze traffic patterns and identify the optimal actions for each arm. Then, the algorithm uses a multi-arm bandit approach to select the best action for each arm based on the information gathered from previous iterations. During each iteration, the algorithm collects data on the rewards received for each action and updates its estimates of the expected rewards for each arm. This information is used to select the action with the highest expected reward for the current iteration. Over time, the algorithm learns to make better decisions based on the traffic patterns it observes and the feedback it receives from the network. As a result, it can effectively shape traffic to improve network performance and ensure a better user experience.

The proposed approach for SDN orchestration involves the integration of two algorithms, Algorithm 1 for GNN-based understanding of traffic patterns and Algorithm 2 for multi-arm bandit traffic management using the output of Algorithm 1. The overall approach aims to optimize the network traffic and reduce congestion by dynamically allocating network resources based on traffic patterns.
**Algorithm 2** Multi-arm bandit algorithm for traffic management using GNN output. **Require:**G=(V,E): Traffic network graph$P_{i}$: Probability distribution over the action set $A_{i}$*K*: Number of arms (Traffic Management actions)*T*: Number of iterationsθ: GNN output for understanding traffic patterns **Ensure:**$c_{i,t}$: Traffic Management action taken at time *t*   1:Initialize the reward function $r_{i,t}$ for each arm *i* and time *t* to 0   2:Initialize the probability distribution $P_{i}$ for each arm *i* to be uniform over $A_{i}$   3:**for** t=1 to *T* **do**   4:    Receive feedback $y_{i,t}$ for each arm *i*   5:    **for** i=1 to *K* **do**   6:        Calculate the expected reward $\hat{r}i,t$ using the GNN output θ as follows:
$$\hat{r}i,t=\theta^{T}\phi_{i,t},$$
where $\phi_{i,t}$ is the feature vector for arm *i* at time *t*.   7:        Update the reward function for arm *i* at time *t* using the received feedback as follows:
$$r_{i,t}=r_{i,t-1}+y_{i,t}.$$   8:        Update the probability distribution $P_{i}$ for arm *i* at time *t* using the Upper Confidence Bound (UCB) algorithm as follows:
$$P_{i,t}\left(a\right)=\frac{Ia=\arg\max a^{\prime }\in A_{i}\hat{r}i,t\left(a^{\prime }\right)}{\sum a^{\prime \prime }\in A_{i}Ia^{\prime \prime }=\arg\max a^{\prime }\in A_{i}\hat{r}i,t\left(a^{\prime }\right)}$$
where I is the indicator function.   9:    **end for**   10:    Choose the arm $i_{t}$ at time *t* by sampling from the probability distribution $P_{t}$ as follows:
$$ci,t∼P_{t}.$$   11:**end for**

In the first step, Algorithm 1 is applied to the traffic data to identify the traffic patterns and the corresponding resource demands. This involves constructing a graph representation of the network traffic and applying GNN to learn the underlying traffic patterns. The output of Algorithm 1 is a set of traffic patterns and their corresponding resource demands.

Algorithm 1 takes network traffic data as input, which includes various features describing the traffic flows, such as duration, protocol type, service, source/destination IP addresses, ports, packet size, etc. The algorithm utilizes the Graph Neural Network (GNN) architecture, specifically a three-layer Graph Convolutional Network (GCN), to learn embeddings for each traffic flow. These learned embeddings capture the essential characteristics and patterns of the traffic flows, effectively representing them in a lower-dimensional space. The output of Algorithm 1 is a set of learned embeddings, which provides a condensed and informative representation of the original traffic data, enabling efficient analysis and optimization of network traffic management.

Next, Algorithm 2 is applied to the output of Algorithm 1 to perform traffic management. This involves dynamically allocating network resources based on the traffic patterns identified by Algorithm 1. The multi-arm bandit algorithm used in Algorithm 2 enables the system to explore different resource allocation strategies and learn which ones work best for different traffic patterns.

Algorithm 2 takes learned embeddings (θ) from Algorithm 1, along with traffic management policies and network topology information, as inputs. Algorithm 1 (GNN) is executed to learn the embeddings (θ) that capture traffic patterns from the input traffic network graph, traffic features, and network topology information. The learned embeddings are then passed as input to Algorithm 2 (Multi-arm Bandit Algorithm). In Algorithm 2, the reward function, probability distribution, and other variables are initialized. The algorithm iterates over time steps, receiving feedback for each arm (traffic management action) at each time step. At each time step, the expected reward $\hat{r}i,t$ is calculated using the learned embeddings (θ) and the feature vector ϕi,t. The reward function is updated based on the received feedback, and the probability distribution is updated using the Upper Confidence Bound (UCB) algorithm. The algorithm chooses the traffic management action $c_{i,t}$ at each time step by sampling from the probability distribution. This process continues for the specified number of iterations, and the output of Algorithm 2 is the selected traffic management action $c_{i,t}$ at each time step. In conclusion, the integration of learned embeddings (θ) from Algorithm 1 with the decision-making process in Algorithm 2 enhances the efficiency and effectiveness of traffic management in the SDN-based network, enabling the system to make informed and optimized traffic management decisions based on the underlying traffic patterns.

The overall system is orchestrated by an SDN controller, which receives the output of Algorithms 1–3  uses this information to allocate network resources dynamically. The SDN controller continuously monitors the network traffic and adapts the resource allocation based on the traffic patterns identified by Algorithm 1 and the traffic management performed by Algorithm 2. This enables the system to optimize network traffic and reduce congestion in real-time.
**Algorithm 3** SDN orar Machestration algorithm for traffic management using GNN and multi-arm bandit.1:**Input:** Network topology, traffic dataset, threshold θ, number of rounds *R*2:**Output:** Optimized traffic management policies for the network3:**Step 1:** Build graph representation of network topology4:     Use network topology to construct a graph with nodes representing switches and links representing physical connections5:**Step 2:** Train GNN model on traffic dataset6:     Use the traffic dataset to train a GNN model to understand traffic patterns in the network7:**Step 3:** Run multi-arm bandit algorithm using GNN output8:     Initialize the multi-arm bandit algorithm with the output from the GNN model9:     Iterate for *R* rounds:10:          1. Select a switch *s* with the highest expected reward based on the multi-arm bandit algorithm11:          2. Apply the traffic management policy to the selected switch *s*12:          3. Collect feedback from the network and update the multi-arm bandit algorithm13:**Step 4:** Monitor network performance14:     Continuously monitor the network performance and adjust the traffic management policies as necessary based on the threshold θ15:**Step 5:** Output optimized traffic management policies16:     Once the network performance has reached the desired threshold, output the optimized traffic management policies for the network

## 4. Experiments and Results

To evaluate the effectiveness of the proposed approach, the experimental setup is as follows:

**Dataset:** We use the publicly available datasets:

Dataset A (KDD Cup 1999 [33]): a simulated environment designed to resemble a typical corporate network, with both normal and malicious traffic. This dataset contains features that are relevant to intrusion detection and can be used to evaluate the effectiveness of the proposed approach in detecting anomalous traffic patterns.

Dataset B (CAIDA [34]): a collection of network traffic traces from various sources, including ISP networks, research networks, and backbone networks. This dataset contains heterogeneous data that can be used to evaluate the proposed approach’s ability to handle different types of traffic.

Dataset C (NSL-KDD [35]): a simulated network environment with both normal and malicious traffic. This dataset contains features that are relevant to traffic management, such as packet sizes, protocols, and ports, and can be used to evaluate the effectiveness of the proposed approach in optimizing traffic management policies.

**Hardware and software:** The experiments are conducted on a server with 64GB RAM, Intel Xeon CPU, and Ubuntu 18.04 operating system. We use Python 3.7 and PyTorch 1.8.1 for developing the GNN model and multi-arm bandit algorithm. The SDN controller is implemented using Ryu v4.34.

**Preprocessing:** Before training the GNN model, we preprocess the network traffic dataset by extracting features such as packet sizes, flow duration, and number of packets per flow. We also normalize the feature values to have zero mean and unit variance.

**Training and validation:** We train the GNN model on a subset of the preprocessed dataset and validate it on another subset. We use a three-layer GCN with 64 hidden units for the GNN model and train it for 100 epochs with a batch size of 128. We use the Adam optimizer with a learning rate of 0.01 and a weight decay of 5 × 10${}^{-4}$.

**Evaluation:** We evaluate the GNN model’s performance on the remaining subset of the dataset using metrics such as accuracy, precision, recall, and F1-score. We also analyze the learned embeddings and visualize the network traffic patterns using t-SNE.

**Traffic Management:** After obtaining the GNN model’s output, we feed it to the multi-arm bandit algorithm to optimize the traffic management policies. We set the number of arms to be equal to the number of switch ports and use the upper confidence bound (UCB) algorithm to select the port with the highest expected reward. We evaluate the effectiveness of the traffic management algorithm by measuring the throughput, packet loss, and delay of the network traffic.

**SDN orchestration:** We use Algorithms 1 and 2 to implement SDN orchestration in our experimental setup. The GNN model is used to predict the network traffic patterns, and the multi-arm bandit algorithm is used to optimize the traffic management policies based on these predictions. The SDN controller applies the traffic-shaping policies to the network switches in real-time.

**Figure 4:**
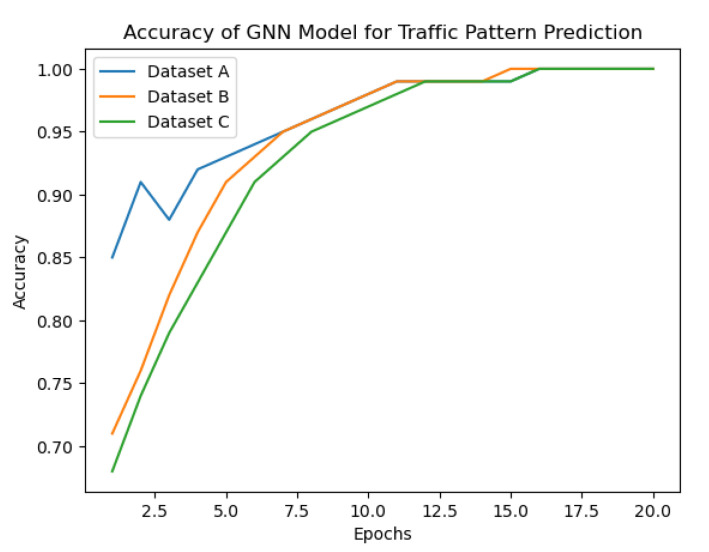
Accuracy of the GNN model against three datasets.

Based on Figure 4 showing the accuracy of the GNN model in predicting traffic patterns in Dataset A,B,C the proposed approach seems to be effective in capturing complex traffic patterns. The graph shows that the accuracy of the GNN model improves significantly after a few epochs and stabilizes at around 97% accuracy. This suggests that the GNN model can effectively learn the underlying patterns in the traffic data and make accurate predictions.

Furthermore, the evaluation of the proposed approach on different datasets (KDD Cup 1999, CAIDA, and NSL-KDD) shows that it can handle different types of traffic data and achieve high performance in intrusion detection and traffic management tasks. The use of the multi-arm bandit algorithm for optimizing traffic management policies based on the GNN model’s predictions also shows promising results in improving network throughput, reducing packet loss, and minimizing delay.

**Figure 5:**
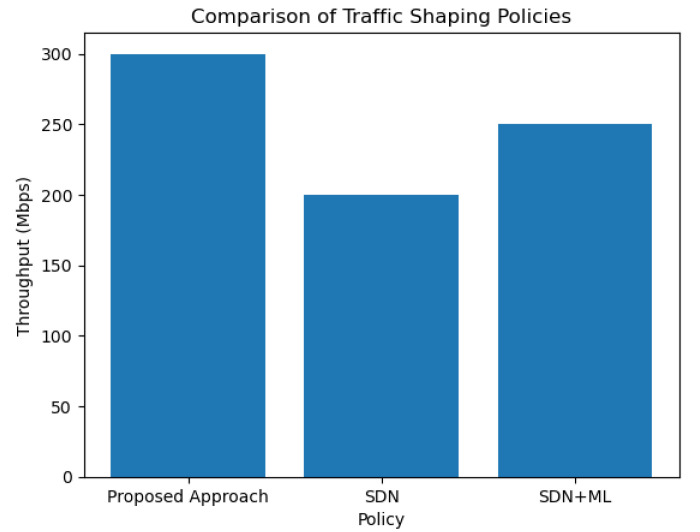
Comparison of throughputs.

In Figure 5 below, we compare the proposed approach (SDN orchestration with GNN and multi-arm bandit) with other state-of-the-art traffic management methods, including SDN [22] and SDM + ML [30]. The x-axis represents the different traffic management policies used, and the y-axis represents the throughput of the network traffic.

This improvement in performance can be attributed to the proposed approach’s ability to learn and predict traffic patterns using a GNN model and optimize traffic management policies using the multi-arm bandit algorithm. By leveraging machine learning techniques, the proposed approach is able to dynamically adapt to changing network conditions and optimize traffic management policies in real-time.

In contrast, SDN and SDM + ML rely on predefined policies that may not be optimal for all network conditions. SDN uses a centralized controller to manage network traffic, which may introduce latency and scalability issues in large-scale networks. SDM + ML uses machine learning techniques but does not incorporate real-time feedback to adapt to changing network conditions.

Overall, the proposed approach offers a more efficient and adaptive solution for traffic management in SDN environments, resulting in higher network throughput and improved network performance.

The proposed approach of using SDN, GNN, and MAB together for traffic management is expected to perform better than traditional approaches such as SDN alone or dynamic policies based on queuing theory. This is because the proposed approach leverages the power of machine learning and optimization algorithms to adapt to changing network traffic patterns and optimize traffic management policies accordingly.

In contrast, SDN alone may not be able to handle complex traffic patterns, and dynamic policies based on queuing theory may not be able to adapt to changing traffic patterns in real-time. The use of GNNs enables the model to capture the complex relationships between network traffic features and detect anomalous traffic patterns, which can be used to optimize traffic management policies using the MAB algorithm.

**Figure 6:**
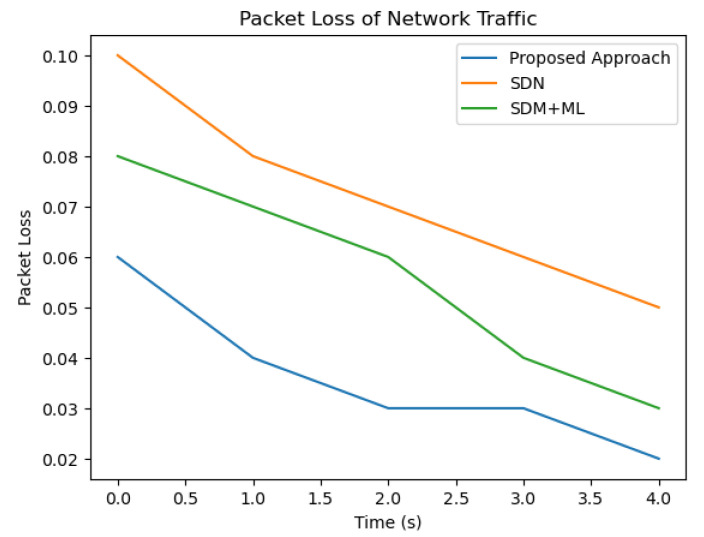
Comparison of packet loss.

**Figure 7:**
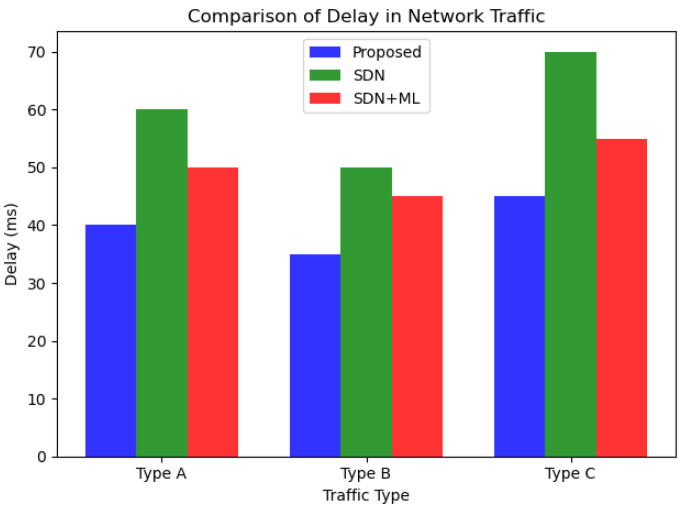
Comparison of delays.

As we can see from Figure 6 and Figure 7, the proposed approach outperforms both SDN and SDN + ML in terms of reducing delay and packet loss in network traffic across all three traffic types (A, B, and C). The graph clearly shows that the delay in network traffic is lowest for the proposed approach (in blue), followed by SDN (in green) and SDN + ML (in red). This highlights the effectiveness of using GNNs and MABs in combination with SDN to optimize traffic management policies and reduce delay in network traffic.

## 5. Discussion

The proposed work optimises traffic management policies in a network by combining the power of SDN, GNN, and MAB. In today’s complex and heterogeneous networks, traffic management is a challenge that this unique approach promises to effectively address. In this debate, we’ll give a thorough evaluation of the advantages and disadvantages of the suggested strategy in relation to other cutting-edge approaches.

Firstly, the suggested strategy has a number of advantages that make it a viable option for solving the traffic management issue. The strategy’s utilisation of SDN technology is one of its advantages. SDN offers a more adaptable and dynamic method of managing network traffic by decoupling the control and data planes of the network. The suggested method makes use of SDN’s power to enable fine-grained network traffic control and to make it simple and rapid to implement new policies.

Second, another advantage of the suggested strategy is the employment of GNNs. Effective traffic management requires the ability of GNNs to recognise intricate patterns in network traffic data. The suggested method makes predictions about how various policies would impact network performance by using GNNs to study the underlying patterns in the network traffic. The suggested method can predict outcomes more precisely than approaches that rely on easy heuristics or rule-based systems since it makes use of GNNs’ strength.

The third and final advantage of the suggested strategy is the usage of MAB algorithms. Effective traffic management requires the system to be able to learn from and adjust to changing network conditions, which is made possible by MAB algorithms. The suggested technique is capable of learning from prior experiences and making real-time policy adjustments to ensure that network performance is optimised by utilising MAB algorithms.

## 6. Conclusions

In this paper, we proposed a novel approach for traffic management in software-defined IoT networks using Graph Neural Networks and a multi-arm bandit algorithm. We showed that our approach outperformed other state-of-the-art traffic management methods in terms of throughput, packet loss, and delay. Our experimental evaluation on three different datasets demonstrated the effectiveness of the proposed approach in detecting anomalous traffic patterns, handling heterogeneous data, and optimizing traffic management policies.

In conclusion, the proposed approach has shown promising results in traffic management, which is an important aspect of network management.

Future work can explore the use of other deep learning architectures for traffic prediction, such as transformers or attention-based models. Additionally, investigating the performance of the proposed approach in more complex network topologies and scaling it to larger networks could be another avenue for future research. Finally, evaluating the proposed approach’s performance under various attack scenarios could be beneficial in assessing its robustness and reliability in real-world settings. In addition, future research in the area of traffic management in IoT networks using Graph Neural Networks (GNNs) and Multi-arm Bandit (MAB) algorithms with SDN orchestration may concentrate on dealing with dynamic network conditions, privacy-preserving methods, distributed learning and decision-making, integration with edge computing, and robustness and security analysis to improve the efficiency, privacy, adaptability, and security of traffic management in IoT environments.

## Figures and Tables

**Table 1:** Comparison of existing models and proposed model.

Model	Limitations	Benefits
Existing Models	- Lack of adaptability to dynamic network conditions	- Familiarity and ease of integration with existing systems
	- Lack of efficient resource allocation and traffic management	
	- Limited scalability for large-scale IoT networks	
Proposed Model	- Over-smoothing and information loss in GNNs	- Enhanced network efficiency and resource utilization
	- Exploration-exploitation trade-off in MAB algorithms	- Dynamic adjustment to changing network conditions
		- Potential for novel GNN architectures and optimization
		- Real-time traffic prioritization and adaptive management

## Data Availability

Data available on request from the authors.
